# The Role of CRISPR/Cas9 in Revolutionizing Duchenne's Muscular Dystrophy Treatment: Opportunities and Obstacles

**DOI:** 10.1055/s-0044-1791803

**Published:** 2024-10-18

**Authors:** Ahsan Ali, Md Yakeen Rahman, Danish Sheikh

**Affiliations:** 1 Faculty of Health, Plymouth University Peninsula Medical School, Plymouth, United Kingdom; 2 University of Plymouth Faculty of Heath, Plymouth, United Kingdom

**Keywords:** CRISPR/Cas9, Duchenne's muscular dystrophy, gene editing, gene therapy, preclinical models

## Abstract

Duchenne's muscular dystrophy (DMD) is a severe X-linked disorder characterized by progressive muscle degeneration, leading to loss of ambulation, respiratory failure, and premature death. It affects approximately 1 in 3,500 live male births and is caused by mutations in the dystrophin gene, which impairs muscle fiber stability. Current treatments are limited to managing symptoms and slowing disease progression, with no curative therapies available. The advent of CRISPR/Cas9 gene-editing technology has introduced a promising approach for directly correcting the genetic mutations responsible for DMD. This review explores the potential of CRISPR/Cas9 as a transformative therapy for DMD, highlighting its successes in preclinical models, the challenges associated with its delivery, and the obstacles to its clinical application. While preclinical studies demonstrate the efficacy of CRISPR/Cas9 in restoring dystrophin expression and improving muscle function, significant hurdles remain, including optimizing delivery methods and ensuring long-term safety.

## Introduction

Duchenne's muscular dystrophy (DMD) is a devastating and progressive neuromuscular disorder caused by mutations in the dystrophin gene (Xp21.2). It is one of the most common genetic conditions, affecting approximately 1 in 3,500 male births worldwide. 1 DMD typically manifests between the ages of 3 and 6 years, with initial symptoms including muscle weakness and wasting (atrophy) in the pelvic area, followed by the involvement of the shoulder muscles. As the disease progresses, muscle weakness extends to the trunk, forearms, and eventually other muscles throughout the body. The calves often appear enlarged, a hallmark of DMD. By the teenage years, most individuals with DMD require a wheelchair, and the disease can lead to severe, life-threatening complications, including cardiomyopathy and respiratory failure. 2 3

**Fig. 1:**
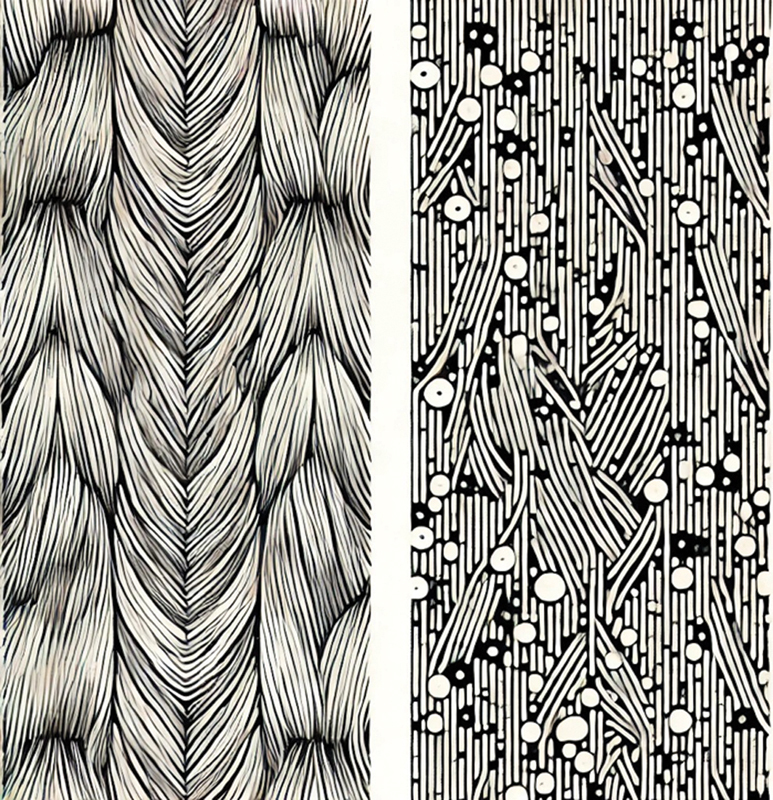
Abstract representation of muscle tissue comparison between healthy individuals and those affected by Duchenne's muscular dystrophy (DMD). On the left, the healthy muscle tissue is depicted as *evenly aligned, organized lines* symbolizing intact muscle fibers. On the right, the DMD-affected muscle tissue shows *irregular, broken lines* , representing the disorganized and degenerating muscle fibers characteristic of DMD progression.

DMD is caused by mutations in the DMD gene, which encodes the dystrophin protein. Dystrophin is a key component of the dystrophin–glycoprotein complex that stabilizes the muscle cell membrane (sarcolemma) during contraction. The absence or severe deficiency of functional dystrophin due to genetic mutations results in continuous muscle damage, inflammation, and fibrosis, ultimately leading to the characteristic muscle weakness and premature death seen in DMD patients. 1 4 The disease is inherited in an X-linked recessive manner, primarily affecting males, although females can be carriers and, in rare cases, manifest mild symptoms ( Fig. 1 ).

Current treatment strategies for DMD focus on managing symptoms and slowing disease progression. Corticosteroids, such as prednisone and deflazacort, are the mainstay of treatment, helping prolong ambulation, preserve respiratory function, and delay scoliosis. However, these benefits come at the cost of significant side effects, including weight gain, bone fragility, and behavioral changes. Physical therapy is essential in maintaining muscle strength and flexibility, while assisted ventilation supports respiratory function in later stages of the disease. 5 Emerging treatments, such as exon-skipping therapies (e.g., eteplirsen), aim to restore the dystrophin reading frame and produce a partially functional dystrophin protein, but these approaches only apply to a subset of patients and do not fully address the underlying genetic cause. 6

Despite these advances, current treatments for DMD remain largely palliative rather than curative, underscoring the need for therapies that address the root cause of the disease. Gene-editing technologies, particularly CRISPR/Cas9, have emerged as a promising approach to directly target and correct the genetic mutations responsible for DMD. CRISPR/Cas9 has the potential not only to alleviate symptoms but also to provide a curative solution by restoring dystrophin expression through precise genetic correction. 4 Recent advancements have demonstrated the feasibility of CRISPR/Cas9-mediated exon skipping and other genome-editing strategies, such as base editing and prime editing, to correct a wide range of DMD mutations, offering broader applicability than traditional approaches.

In preparing this review, a comprehensive search of relevant literature was conducted, focusing on studies related to the application of CRISPR/Cas9 technology in DMD. The literature was identified using databases such as PubMed and PubMed Central (PMC), with an emphasis on publications that provided significant insights into the mechanisms, preclinical models, and therapeutic implications of CRISPR/Cas9 in DMD. Selection criteria prioritized studies that were highly relevant to the topic, excluding those that focused solely on other types of muscular dystrophy or were not written in English.

While preclinical studies have laid a solid foundation for the potential use of CRISPR/Cas9 in treating DMD, recent advancements in human trials for other genetic diseases, such as sickle cell disease and beta-thalassemia, have demonstrated the feasibility and safety of gene-editing technologies. These successes pave the way for future clinical trials of CRISPR/Cas9 specifically targeting DMD patients.

This review evaluates the potential of CRISPR/Cas9-based gene therapy for DMD by exploring recent advancements in the field, addressing the challenges associated with delivery mechanisms, and considering the prospects for clinical application. Additionally, it will discuss the ethical implications of such a transformative therapy, which has the potential to fundamentally change the treatment landscape for DMD and other genetic disorders.

## Mechanism of CRISPR/Cas9 Gene Editing

**Fig. 2:**
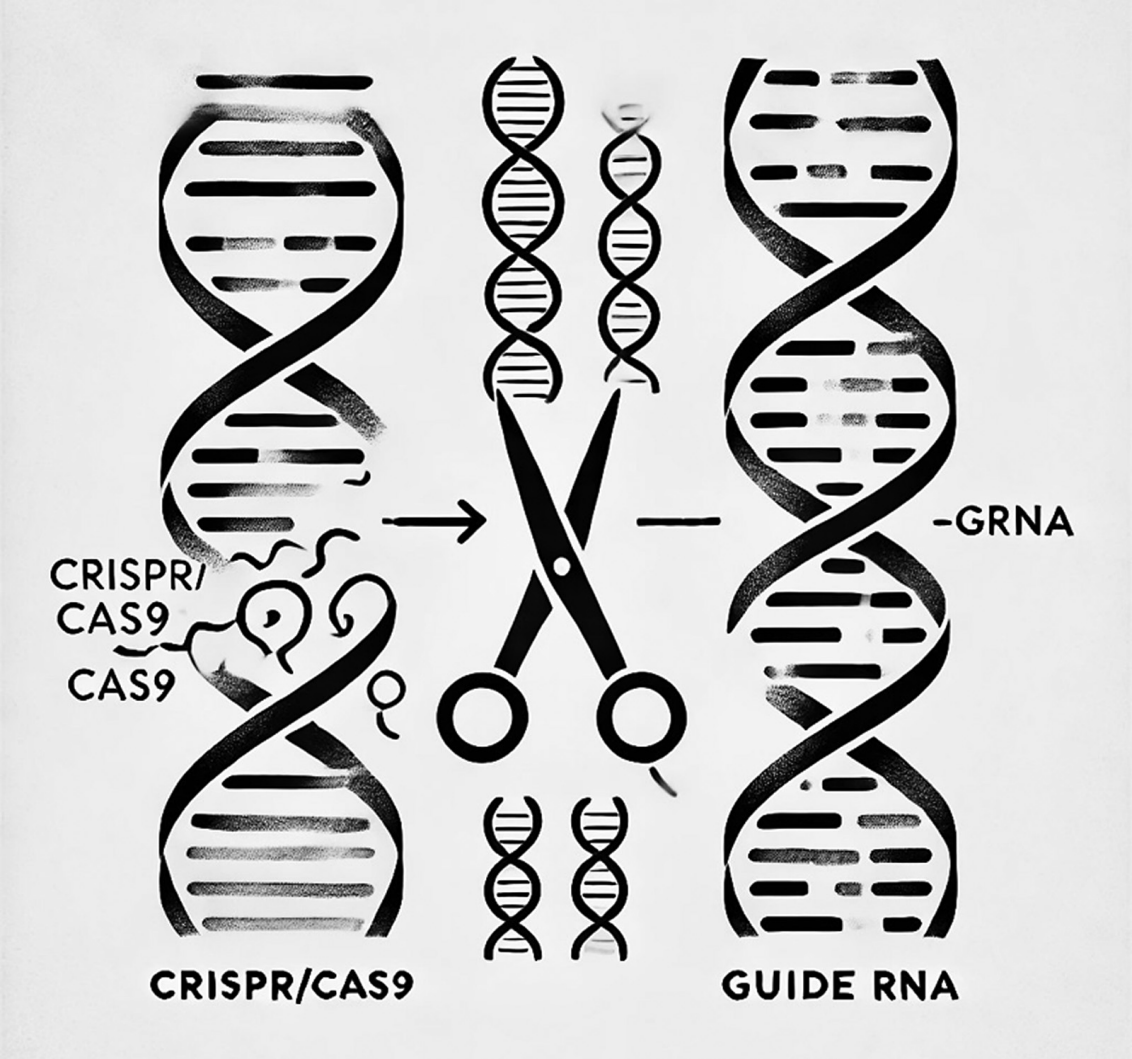
Abstract depiction of the basic CRISPR/Cas9 gene-editing mechanism. On the left, a section of mutated deoxyribonucleic acid (DNA) is represented by a *broken line* . In the center, the CRISPR/Cas9 complex, symbolized by *scissors* , targets the mutation. On the right, the repaired DNA is shown as a *continuous, smooth line* , representing successful gene correction.

CRISPR/Cas9 is a revolutionary tool for genome editing, enabling precise modifications by harnessing natural deoxyribonucleic acid (DNA) repair mechanisms. The system uses a guide RNA (gRNA) to direct the Cas9 enzyme to a specific DNA sequence, where the enzyme introduces a double-stranded break (DSB). 7 8 This break activates repair pathways like nonhomologous end joining (NHEJ), which is error prone and often causes small insertions or deletions, and homology-directed repair (HDR), which can precisely repair the break using a DNA template. 9 In DMD, CRISPR/Cas9 can target and correct mutations in the dystrophin gene, restoring the production of functional dystrophin protein ( Fig. 2 ).

## Next-Generation CRISPR Systems: Cas12a and Enhanced Safety

Recent advancements, such as Cas12a (Cpf1), have expanded the flexibility of CRISPR systems. Cas12a recognizes different protospacer adjacent motif (PAM) sequences, creates staggered cuts, and requires only a single RNA molecule, making it a valuable alternative for certain applications. 10 Additionally, high-fidelity Cas9 variants (e.g., eSpCas9, SpCas9-HF1) and optimized gRNA design have significantly reduced off-target effects, improving the precision of CRISPR-based therapies. 9 11

## Innovative Genome Editing: Base Editing and Prime Editing

New techniques, such as base editing and prime editing, offer more precise and efficient alternatives for correcting DMD mutations. Base editing allows for direct base pair conversions without creating DSBs, 12 while prime editing uses Cas9 nickase and reverse transcriptase to introduce a wider range of edits, including insertions and deletions. 13 14 Both methods have shown potential in restoring dystrophin production in DMD models.

## Therapeutic Implications of Advanced Genome Editing

The development of base- and prime-editing technologies has opened new avenues for DMD therapy, enabling highly targeted corrections with fewer off-target effects. These approaches hold promise for addressing a broader spectrum of mutations with greater precision and safety. 15

Table 1 summarizes the key CRISPR/Cas9 gene-editing approaches for DMD, their mechanisms, and key outcomes.

**Table 1:** Summary of CRISPR/Cas9 gene-editing approaches for DMD, their mechanisms, and key outcomes

Gene-editing approach	Description	Key findings/outcome	Study
Exon skipping	CRISPR/Cas9 targets specific exons to restore the reading frame, producing a shortened but functional dystrophin protein	Demonstrated efficacy in animal models such as the Mdx mouse, restoring dystrophin expression and improving muscle function	Min et al 4
Base editing	Converts specific DNA bases (A-to-G or C-to-T) without creating double-stranded breaks, ideal for correcting point mutations	Shown to correct point mutations in preclinical models, providing a more precise therapeutic option with reduced off-target effects	Rees and Liu 12
Prime editing	Combines Cas9 nickase and reverse transcriptase to perform precise edits, including point mutations, insertions, and deletions	Demonstrated versatility in correcting various mutations in DMD models, with long-term dystrophin restoration and improved muscle function	Anzalone et al 13 and Ryu et al 14

Abbreviations: DNA, deoxyribonucleic acid; DMD, Duchenne's muscular dystrophy.

## Summary of CRISPR/Cas9 Mechanisms and Recent Progress

In summary, CRISPR/Cas9 and its next-generation variants, including base and prime editing, represent a promising frontier in the treatment of DMD. 16 These tools offer increasingly precise and efficient means to correct genetic mutations, with reduced risks of off-target effects and enhanced safety. The ongoing development of these technologies holds the potential to transform therapeutic approaches to DMD, moving closer to long-term, personalized genetic treatments. 4

## Comparative Effectiveness in Preclinical Models for DMD

Preclinical studies have consistently demonstrated that CRISPR/Cas9 can effectively correct dystrophin mutations in animal models of DMD. In a landmark study published in *Science* (2014), CRISPR/Cas9 was used to excise a defective exon from the dystrophin gene in a mouse model of DMD, leading to the expression of a shortened but functional dystrophin protein. Treated mice exhibited improved muscle strength and reduced pathology compared to untreated controls. 17 18

Further research expanded on these findings by demonstrating the potential of CRISPR/Cas9 in larger animal models, including dogs and pigs. A study published in *Molecular Therapy* (2017) reported systemic delivery of CRISPR/Cas9 using adeno-associated virus (AAV) vectors in a canine model, restoring dystrophin expression in various muscles, including the heart, a key target given the cardiomyopathy associated with DMD. 2 Similarly, pigs were used to model DMD due to their physiological similarity to humans, with CRISPR/Cas9 successfully restoring dystrophin expression across multiple muscle types. 19

Table 2 summarizes key studies on DMD animal models generated using the CRISPR-Cas system.

**Table 2:** Key studies on DMD animal models generated using the CRISPR-Cas system

Animal model	Description	Key findings	Study
Mdx mouse model	Mice harboring a mutation in exon 23 of the dystrophin gene, used extensively for DMD research due to its genetic similarity to human DMD, albeit with a milder phenotype	CRISPR/Cas9-mediated exon excision led to partial restoration of dystrophin in skeletal muscles, with improved muscle function. Demonstrated in multiple studies, including early somatic correction. Systemic delivery via AAV vectors increased dystrophin expression and improved strength in skeletal muscle	Munshi, 8 Long et al, 17 and Nelson et al 20
Canine model (deltaE50-MD)	Dogs with exon 50 deletion in the dystrophin gene, closely replicating the human form of DMD, including muscle degeneration and cardiomyopathy	Systemic CRISPR/Cas9 delivery via AAV vectors restored dystrophin expression across multiple muscles, including the heart. Restoration levels varied across muscle groups (3–90%). Higher doses showed significant functional improvements, particularly in cardiac muscle	Chamberlain and Chamberlain, 2 Mata López et al, 6 and Amoasii et al 18
Pig model	Pigs engineered with dystrophin mutations using CRISPR/Cas9, providing a larger, more physiologically relevant model for human DMD	CRISPR/Cas9 corrected dystrophin mutations via AAV9 vectors, leading to widespread dystrophin expression in skeletal, diaphragm, and cardiac muscles. The treatment improved muscle function and extended survival rates, addressing key challenges related to long-term efficacy and immune responses	Hoffman et al 3 and Moretti et al 19

Abbreviations: AAV, adeno-associated virus; DMD, Duchenne's muscular dystrophy.

## Comparative Analysis of Preclinical Models

The use of animal models has been instrumental in demonstrating proof-of-concept for CRISPR/Cas9-mediated gene correction in DMD. The mouse model has provided invaluable insights into the feasibility of CRISPR-mediated exon excision, but its mild phenotype limits its direct translation to human DMD. 8 Canine models, such as the deltaE50-MD dog, offer a closer approximation of human disease, particularly in terms of cardiac involvement, although variability in therapeutic outcomes and immune responses remain challenges. 6 18 Pig models, with their closer physiological resemblance to humans, offer a robust platform for assessing long-term efficacy and safety, although they are more expensive and logistically challenging. 19

Moving forward, the results from these preclinical studies will be critical in guiding the design of human trials, ensuring that delivery methods and dosages are optimized to achieve consistent, long-lasting dystrophin restoration across all affected muscles.

## Clinical Translation of CRISPR/Cas9: Human Trials for Other Genetic Diseases

The successful application of CRISPR/Cas9 in treating genetic diseases has moved from preclinical models to early-stage human trials. Notably, clinical trials have been initiated to correct genetic mutations in conditions like sickle cell disease and beta-thalassemia. 21 22 These trials involve editing hematopoietic stem cells ex vivo to reintroduce them into the patient's body, aiming to produce healthy blood cells and mitigate disease symptoms. For instance, a 2021 study demonstrated that CRISPR/Cas9-mediated gene editing led to the sustained production of fetal hemoglobin in sickle cell patients, thereby alleviating the severity of their symptoms. 21 22 These early human trials underscore the therapeutic potential of CRISPR/Cas9 and provide critical safety and efficacy data, forming the groundwork for applying similar strategies to other genetic disorders, including DMD.

The positive outcomes from these trials indicate that CRISPR/Cas9 can achieve precise and lasting genetic modifications with a manageable safety profile. This advancement holds particular promise for the treatment of DMD, suggesting a viable path toward clinical trials specifically targeting the dystrophin gene. The experience gained from ongoing human trials will be invaluable in designing clinical strategies for DMD, potentially accelerating the timeline for bringing CRISPR-based therapies to patients suffering from this debilitating disease.

## In Vivo Delivery Approaches: Challenges and Innovations in CRISPR/Cas9 Delivery for DMD

One of the major challenges in using CRISPR/Cas9 therapy for DMD is effectively delivering the gene-editing components to muscle cells. The large size of the dystrophin gene and the CRISPR components, including Cas9 and gRNA, often exceeds the packaging capacity of AAV vectors, 4 which are commonly used for gene delivery due to their low immunogenicity and tissue tropism. 5 23

Various strategies have been developed to overcome these delivery limitations, ranging from the use of smaller Cas proteins to nonviral delivery systems. These innovations have greatly enhanced the potential of CRISPR/Cas9 to target dystrophin mutations effectively.

Table 3 summarizes the key strategies developed to overcome the delivery limitations in CRISPR/Cas9 therapy for DMD.

**Table 3:** Key strategies developed to overcome delivery limitations in CRISPR/Cas9 therapy for DMD

Strategy	Description	Key findings/outcome	Study
Cas12a and dual AAV vectors	Cas12a, a smaller Cas protein, allows for more compact gene-editing components, making it easier to deliver using AAV vectors. Dual AAV vectors can package Cas9 and guide RNA separately to bypass size constraints	Demonstrated successful dystrophin restoration in iPSCs and increased versatility of delivery in models such as neonatal mice	Maggio et al 23 and Zhang et al 24
Multiplex editing	Multiple guide RNAs (gRNAs) are used to target several exons simultaneously, increasing the efficiency of gene correction in the cases involving multiple mutations	Studies have shown that multiplex editing can restore dystrophin expression across multiple muscle groups, with promising results in preclinical models of DMD	Echigoya et al 25 and Nakanishi et al 26
Optimized Cas variants	Cas9 nickase (Cas9n) paired with multiple gRNAs improves specificity, requiring two adjacent target sites to reduce off-target effects and improve safety	These optimized Cas variants, along with chemically modified gRNAs, have been shown to reduce off-target activity while maintaining editing efficiency, particularly in muscle tissues	Nakanishi et al 26
Lipid nanoparticles (LNPs)	Nonviral delivery method using LNPs to encapsulate CRISPR/Cas9 components, facilitating gene editing with reduced immune responses	LNPs have successfully delivered CRISPR/Cas9 ribonucleoprotein complexes to muscle cells, demonstrating effective editing without strong immune responses, paving the way for repeated treatments in chronic conditions	Mukai et al 27 and Vavassori et al 28
Exosome-based delivery	Exosomes, naturally occurring vesicles, can encapsulate and deliver CRISPR/Cas9 components across biological barriers with high specificity and low immunogenicity	Exosome-mediated delivery has been shown to achieve targeted gene editing in muscle tissues with fewer off-target effects, offering a biocompatible and efficient alternative for delivery	Wang et al 29
AAV variants	Novel AAV capsids have been engineered to enhance tissue specificity, evade preexisting antibodies, and increase the capacity for delivering larger gene-editing machinery	Next-generation AAV variants have demonstrated improved muscle targeting and reduced immunogenicity, offering a promising solution for long-term CRISPR/Cas9 delivery in DMD therapy	Nelson et al 30

Abbreviations: AAV, adeno-associated virus; DMD, Duchenne's muscular dystrophy; iPSC, induced pluripotent stem cell; gRNA, guide ribonucleic acid.

### Challenges in Large-Scale Production and Distribution

Another major hurdle that is considered is the large-scale production and distribution of AAV vectors for systemic delivery, which must be sufficient to target the widespread muscle tissues affected by DMD. The dystrophic muscle environment, characterized by degeneration, necrosis, and inflammation, further complicates effective gene transfer and vector persistence. For instance, while AAV vectors were shown to persist in cardiac muscle for up to a year in some studies, their presence in skeletal muscle significantly diminished over time, likely due to promoter silencing or vector genome loss. 30 Ensuring long-term dystrophin expression across all affected tissues remains a critical challenge for the future development of CRISPR-based therapies for DMD.

## Advances in Delivery Systems and Multiplex Editing for DMD

Overall, the progress in optimizing delivery systems, combined with advances in multiplex editing and increased specificity of gene-editing tools, marks a significant step forward in the application of CRISPR/Cas9 therapy for DMD. These developments not only offer solutions to overcome current delivery limitations but also pave the way for more precise and effective treatments. Future research will need to focus on refining these methods to ensure safe, efficient, and widespread delivery, particularly in targeting critical muscle groups such as the heart and diaphragm. As these strategies continue to evolve, they hold the potential to revolutionize the management of DMD and similar genetic disorders.

## Ex Vivo Delivery Approaches for DMD: Patient-Specific iPSCs for Personalized Therapy

Ex vivo gene editing represents a promising and personalized approach to treating DMD. This method involves the genetic correction of patient-derived cells, which are then reintroduced into the patient to repair and regenerate muscle tissue.

A key technology in this approach is the use of induced pluripotent stem cells (iPSCs), which are derived from a patient's somatic cells and reprogrammed to an embryonic-like state.

These iPSCs can be genetically modified using CRISPR/Cas9 to correct mutations in the dystrophin gene, the primary genetic defect in DMD. 31 Once corrected, iPSCs are differentiated into myogenic precursor cells, which are progenitor cells capable of developing into muscle tissue. These precursor cells can be transplanted back into the patient, where they fuse with existing muscle fibers, contributing to muscle repair and regeneration. This method not only addresses the underlying genetic defect but also provides a renewable source of cells that can continuously participate in muscle regeneration.

## Recent Advances in Ex Vivo Gene Editing

In recent years, significant advancements in ex vivo gene editing have been made, particularly in the context of DMD. A landmark study published in 2024 explored the potential of CRISPR/Cas9-mediated gene correction in patient-specific iPSCs. The researchers successfully corrected a mutation in the dystrophin gene in iPSCs derived from a DMD patient. After gene correction, the iPSCs were differentiated into myogenic precursor cells, which were subsequently transplanted into a DMD mouse model. 31 The results of this study were highly promising. The transplanted cells not only survived but also integrated into the existing muscle tissue, where they began to express functional dystrophin protein.

Importantly, these corrected cells contributed to muscle fiber repair and exhibited the potential for long-term muscle regeneration, a critical factor in addressing the progressive muscle degeneration characteristic of DMD.

Moreover, the study highlighted the efficiency and precision of the CRISPR/Cas9 system in correcting single-point mutations in the dystrophin gene, demonstrating minimal off-target effects. This finding is crucial, as it addresses one of the main concerns associated with CRISPR/Cas9—its potential for unintended genomic alterations.

## Advancing toward a Cure: The Role of iPSCs in DMD Therapy

The development of ex vivo gene editing using patient-specific iPSCs marks a significant step forward in the quest for a cure for DMD. The success of these studies not only demonstrates the feasibility of this approach but also highlights its potential for clinical translation. As research progresses, this personalized therapy could complement or even replace existing treatments, providing a more effective and sustainable solution for managing and potentially curing DMD.

## General Ethical Considerations for CRISPR-Based Therapies

The advent of gene-editing technologies like CRISPR/Cas9 presents profound ethical questions, particularly when considering their application in treating genetic disorders such as DMD. While the current focus is on somatic cell editing—where changes are confined to the treated individual and are not passed on to future generations—the potential for germline editing, which would affect descendants, has ignited considerable debate in both scientific and public spheres. The use of CRISPR/Cas9 for DMD would involve editing the dystrophin gene in somatic cells, theoretically eliminating the risk of transmitting edited genes to offspring. 32 However, even somatic gene editing raises significant ethical considerations.

One of the primary ethical concerns is the issue of accessibility. The development and implementation of CRISPR-based therapies are likely to be expensive, which could exacerbate existing disparities in health care access. If such treatments are only available to those who can afford them, this could widen the gap between different socioeconomic groups, leading to a situation where only the wealthy benefit from the latest medical advancements. This raises questions about fairness and justice in the distribution of health care resources, as well as the responsibility of governments and health care systems to ensure equitable access to these potentially life-saving therapies.

Another critical ethical issue is the potential for misuse of CRISPR/Cas9 technology, particularly in the context of human enhancement. While the primary goal of CRISPR research is to treat or prevent serious diseases, there is a concern that the technology could be used for nontherapeutic purposes, such as enhancing physical abilities, intelligence, or appearance. This possibility raises a host of ethical dilemmas, including the potential to create a society divided between those who are genetically “enhanced” and those who are not. Such a scenario could lead to new forms of inequality and discrimination, challenging the very notion of what it means to be human. 33

The socioeconomic impact of gene-editing technologies also warrants careful consideration. As CRISPR-based therapies become more prevalent, there may be broader societal implications, such as changes in population demographics if genetic diseases are eradicated or significantly reduced. Additionally, the potential for gene editing to alter the course of human evolution is a topic of concern, as unintended consequences could arise from the introduction of edited genes into the population.

Furthermore, public perception of gene editing is another crucial factor that influences the ethical landscape. The general public's understanding of CRISPR technology is still developing, and there is a wide range of opinions on its use. Some view CRISPR with optimism, seeing it as a revolutionary tool that could eliminate suffering caused by genetic diseases. Others are more cautious, concerned about the potential risks and ethical implications. Engaging the public in informed discussions about the benefits and risks of CRISPR is essential for fostering a balanced and ethical approach to its use. 33

The regulatory landscape for gene editing is evolving but remains complex and inconsistent across different countries. In some regions, there is a strong regulatory framework that governs the use of CRISPR in both research and clinical settings, ensuring that ethical considerations are thoroughly evaluated before any gene-editing procedures are approved. However, in other regions, regulations may be less stringent, raising concerns about the possibility of unregulated or unethical use of the technology. International collaboration and the development of global standards for the ethical use of CRISPR are critical to addressing these concerns and ensuring that the technology is used responsibly. 34

In summary, while CRISPR/Cas9 offers unprecedented opportunities for treating genetic diseases like DMD, it also raises significant ethical challenges that must be carefully navigated. These include issues of accessibility, potential misuse, socioeconomic impact, public perception, and the need for robust regulatory oversight. As the technology continues to advance, it is essential that the scientific and medical communities, along with policymakers and the public, engage in ongoing dialog to ensure that CRISPR is used in a way that is ethical, equitable, and beneficial for all.

## Potential for Clinical Translation

The preclinical success of CRISPR/Cas9 in DMD models lays a solid foundation for future clinical trials, offering hope for a one-time curative treatment. The ability to correct dystrophin mutations at the genomic level could halt or even reverse the progression of DMD, marking a significant advancement over current therapeutic options.

However, translating these findings into effective human therapies involves overcoming several critical challenges. A primary concern is the delivery of CRISPR/Cas9 components to all affected muscle tissues. Although AAV vectors have demonstrated efficacy in animal models, their application in humans carries risks such as immune responses and challenges in achieving widespread delivery across large muscle groups. 4 7 Despite progress in developing alternative delivery methods, such as nanoparticle-based systems and nonviral vectors, these approaches have yet to match the efficiency of viral vectors in clinical settings.

Another significant challenge is ensuring the long-term safety of CRISPR/Cas9-based therapies. Off-target effects, where CRISPR/Cas9 may unintentionally edit other parts of the genome, could have serious consequences, including the potential for oncogenesis. 29 Advances in gRNA design and high-fidelity Cas9 variants have reduced these risks, but rigorous testing in clinical trials is essential to confirm the safety and efficacy of these therapies. 17 Addressing these delivery and safety challenges is crucial for the successful translation of CRISPR/Cas9 therapies from bench to bedside, paving the way for clinical trials specifically targeting the dystrophin gene in DMD patients.

As research continues to refine these technologies, the lessons learned from ongoing human trials in other genetic diseases, such as sickle cell disease and beta-thalassemia, will be invaluable. These trials provide critical safety and efficacy data that can inform the design of DMD-specific strategies, potentially accelerating the timeline for bringing CRISPR-based therapies to patients.

## Future Directions

The future of CRISPR/Cas9 therapy for DMD hinges on several key research priorities. First and foremost, optimizing delivery methods to achieve efficient and widespread distribution of CRISPR/Cas9 components across all affected muscle tissues remains paramount. The development of novel delivery platforms, such as lipid nanoparticles (LNPs), exosome-based systems, and engineered AAV variants, holds significant promise for overcoming the current limitations of viral vectors. 20 These approaches could offer more precise and targeted delivery mechanisms, enhancing the safety and efficacy of CRISPR/Cas9 therapies for DMD.

Advancements in gRNA design are also critical. More efficient gRNA designs that minimize off-target effects are essential for ensuring greater precision in gene editing, reducing the potential risks associated with unintended genetic modifications. High-fidelity Cas9 variants and improved gRNA algorithms are likely to play a pivotal role in this area, ensuring that CRISPR/Cas9 can be applied safely in clinical settings.

Long-term safety and efficacy remain critical areas of investigation. Comprehensive studies using larger animal models, particularly those that closely mimic human physiology, will be essential to fully understand the implications of CRISPR/Cas9 treatment over extended periods. These studies should include rigorous monitoring for potential immune responses, off-target effects, and the durability of dystrophin expression in treated tissues. The development of robust strategies for long-term patient monitoring is crucial to ensure the sustained success of CRISPR/Cas9 therapies in humans.

Beyond DMD, the adaptability of the CRISPR/Cas9 system opens new avenues for treating a broad spectrum of genetic disorders, particularly those caused by single-gene mutations. As this technology advances, it is poised to become a cornerstone of precision medicine, offering tailored treatments based on individual genetic profiles. 35 However, translating CRISPR/Cas9 into routine clinical practice will require overcoming several regulatory hurdles, including demonstrating the therapy's safety, efficacy, and ethical considerations in human trials. The challenges of scaling production, ensuring consistent delivery, and maintaining long-term safety in diverse patient populations must also be addressed.

Looking ahead, the continued evolution of CRISPR/Cas9, coupled with advancements in delivery systems and genetic editing precision, could revolutionize the treatment landscape for DMD and numerous other genetic diseases. 2 This progress promises a future where genetic disorders are not merely managed but potentially cured, transforming the lives of patients worldwide. However, achieving this vision will require sustained collaboration across the scientific, clinical, and regulatory communities to ensure that CRISPR/Cas9 therapies are safe, effective, and accessible to all who need them.

## Conclusions

CRISPR/Cas9 technology offers a revolutionary approach to treating DMD by directly targeting the genetic mutations underlying the disease.

Significant progress in preclinical models has demonstrated the potential to restore dystrophin expression and improve muscle function, laying a robust foundation for future clinical applications. These advancements signal a shift from symptomatic management to a curative strategy, with the potential to alter the course of DMD therapy profoundly.

Despite these promising developments, several critical challenges persist. Achieving efficient and safe delivery of CRISPR components to all affected muscle tissues, including the heart and diaphragm, remains a primary concern. Recent innovations, such as smaller Cas variants, dual AAV vector systems, and nonviral delivery platforms like nanoparticles and exosomes, are showing promise in overcoming these obstacles. Additionally, advances in multiplex editing and the development of high-fidelity Cas9 variants and optimized gRNAs have significantly enhanced the precision and safety of genome editing, reducing off-target effects and mitigating immune responses.

The therapeutic potential of CRISPR/Cas9 extends beyond correcting single mutations to addressing complex genomic alterations. This capability offers hope for personalized treatments tailored to individual genetic profiles, potentially enabling more comprehensive correction of the diverse mutations present in DMD. The integration of CRISPR/Cas9 with emerging technologies such as base editing and prime editing further expands the possibilities for precise genetic corrections, not only for DMD but also for a broad range of genetic disorders.

As we approach clinical implementation, it is crucial to address the ethical and societal implications of genome editing. Ensuring equitable access to these therapies, maintaining rigorous safety standards, and establishing robust ethical governance frameworks will be essential. Public engagement, transparency in research practices, and the inclusion of diverse stakeholder perspectives will play a vital role in navigating these challenges.

In conclusion, the advancements in CRISPR/Cas9 technology mark the beginning of a transformative era in genetic medicine. By refining delivery methods, enhancing genome-editing precision, and addressing ethical considerations, CRISPR/Cas9 holds the potential to revolutionize the treatment of DMD and other genetic disorders. The continued commitment to innovation, collaboration, and ethical responsibility will be key in realizing the full potential of CRISPR/Cas9, offering renewed hope to patients and their families worldwide.
